# Melatonin stabilizes atherosclerotic plaques: an association that should be clinically exploited

**DOI:** 10.3389/fmed.2024.1487971

**Published:** 2024-12-11

**Authors:** Russel J. Reiter, Ramaswamy Sharma, Alejandro Romero, Fedor Simko, Alberto Dominguez-Rodriguez, Daniel P. Cardinali

**Affiliations:** ^1^Department of Cell Systems and Anatomy, UT Health San Antonio, Long School of Medicine, San Antonio, TX, United States; ^2^Applied Biomedical Sciences, University of the Incarnate Word, School of Osteopathic Medicine, San Antonio, TX, United States; ^3^Department of Pharmacology and Toxicology, Faculty of Veterinary Medicine, Complutense, University of Madrid, Madrid, Spain; ^4^Institute of Pathophysiology, Faculty of Medicine, Comenius University, Bratislava, Slovakia; ^5^Servicio de Cardiología, Hospital Universitario de Canarias, Santa Cruz de Tenerife, Spain; ^6^CENECON, Faculty of Medical Sciences, Universidad de Buenos Aires, and Pontificia Universidad Catolica Argentina, Buenos Aires, Argentina

**Keywords:** atherosclerotic plaque, intraplaque hemorrhage, plaque rupture, macrophage polarization, inflammation, adhesion molecules, heart attack

## Abstract

Atherosclerosis is the underlying factor in the premature death of millions of humans annually. The cause of death is often a result of the rupture of an atherosclerotic plaque followed by the discharge of the associated molecular debris into the vessel lumen which occludes the artery leading to ischemia of downstream tissue and to morbidity or mortality of the individual. This is most serious when it occurs in the heart (heart attack) or brain (stroke). Atherosclerotic plaques are classified as either soft, rupture-prone, or hard, rupture resistant. Melatonin, the production of which diminishes with age, has major actions in converting soft to hard plaques. Experimentally, melatonin reduces the ingrowth of capillaries from the tunica media into the plaque relieving pressure on the plaque, reducing intraplaque hemorrhage and limiting the size of the necrotic core. Moreover, melatonin promotes the formation of collagen by invading vascular smooth muscle cells which strengthen the plaque crown making it resistant to rupture. Melatonin is also a powerful antioxidant and anti-inflammatory agent such that is reduces oxidative damage to tissues associated with the plaque and limits inflammation both of which contribute to plaque cap weakness. Additional benefits of melatonin relative to atherosclerosis is inhibition of adhesion molecules on the endothelial cell surface, limiting the invasion of monocytes into the arterial intima, and reducing the conversion of anti-inflammatory M2 macrophages to pro-inflammatory M1 macrophages. Given the high physiological and financial cost of cardiac and neural ischemic events, this information should be given high priority in the clinical setting.

## 1 Introduction

Atherothrombotic events are strongly correlated with the total extent of cardiovascular atherosclerosis, with these processes contributing to an elevated risk of premature death worldwide. These lipid-driven pathological processes are accompanied by the accumulation of oxidized lipoproteins (oxLDL) and inflammatory cells in the subendothelial intima of the aorta and other major arteries. When these deposits progress to mature atherosclerotic plaques in the coronary arteries, they can rupture, leading to fatal pathological events such as a myocardial infarction. In addition to oxidized lipoprotein deposition in the subendothelial space, inflammation is a major pathological feature in all phases of plaque maturation which results in their gradual deterioration and eventual breakdown. Adverse cardiovascular accidents are often associated with systemic immune-related inflammation. As elsewhere, inflammatory responses in atherosclerotic plaques induce, among other destructive changes, oxidative stress-mediated cellular damage which contributes to necrotic core enlargement and the increased likelihood of plaque rupture. In particular, the activation of inflammasomes, which are inhibited by melatonin, are protein complexes that promote the secretion of proinflammatory cytokines that mediate free radical generation (1).

When a vulnerable plaque bursts, the released debris can interrupt the blood flow and oxygen/glucose supply, and even if only transitory, this causes a devastatingly damaging ischemic episode in the downstream tissue. This is especially serious when it occurs in a coronary artery (or in the brain vessel) where the residual consequences may persist permanently due to the failure of cardiomyocytes and neurons to replenish themselves after such an episode, with the resulting lesion contributing to a reduction of life quality and often premature death.

## 2 The atherosclerotic plaque: melatonin inhibition

To secure the available appropriate literature related to the subject, the conventional biomedical data bases including PubMed, Web of Science, and Scopus were searched up to August 2024 using the following terms in various combinations: melatonin, atherosclerosis, atherosclerotic plaque, plaque structure, soft plaque, hard plaque, plaque rupture, plaque fiber cap, plaque calcification, macrophages, adhesion molecules, blood vessel invasion, internal bleeding, heart attack, cardiac ischemia/reperfusion; necrotic core, foam cells; inflammation, lumen stenosis, vessel wall thickness. The searches extended back to 1980 or before.

Vascular smooth muscle (VSM) cells are major constituents of the blood vessel wall and have a significant impact on the maintenance of the morphophysiological integrity of these structures. VSM cells display an uncommon phenotypic plasticity which aids their involvement in pathologies of the vascular system (2). VSM cells are typically of the contractile phenotype but are otherwise relatively quiescent with a low rate of cell division. These cells become highly activated during plaque development when they divide and transform into a different cell type. When doing so, the cells convert to a highly proliferative and inflammation-producing cell type which is highly injurious to atherosclerotic plaque stability.

The molecular transcription factors that support the phenotypic switching of VSM cells during atherosclerosis have been partially identified (3). Initially, KLF4 (Kruppel-like factor 4) reduces the major expression of a gene involved in smooth muscle contraction by suppressing SRF (serum response factor) (4); this gene is essential not only for smooth muscle contraction but also for cytoskeletal functions. Preventing the conversion of smooth muscles cells to the new phenotype interferes with the progression of atherosclerotic lesions and, importantly, contributes to plaque stability.

**Figure 1:**
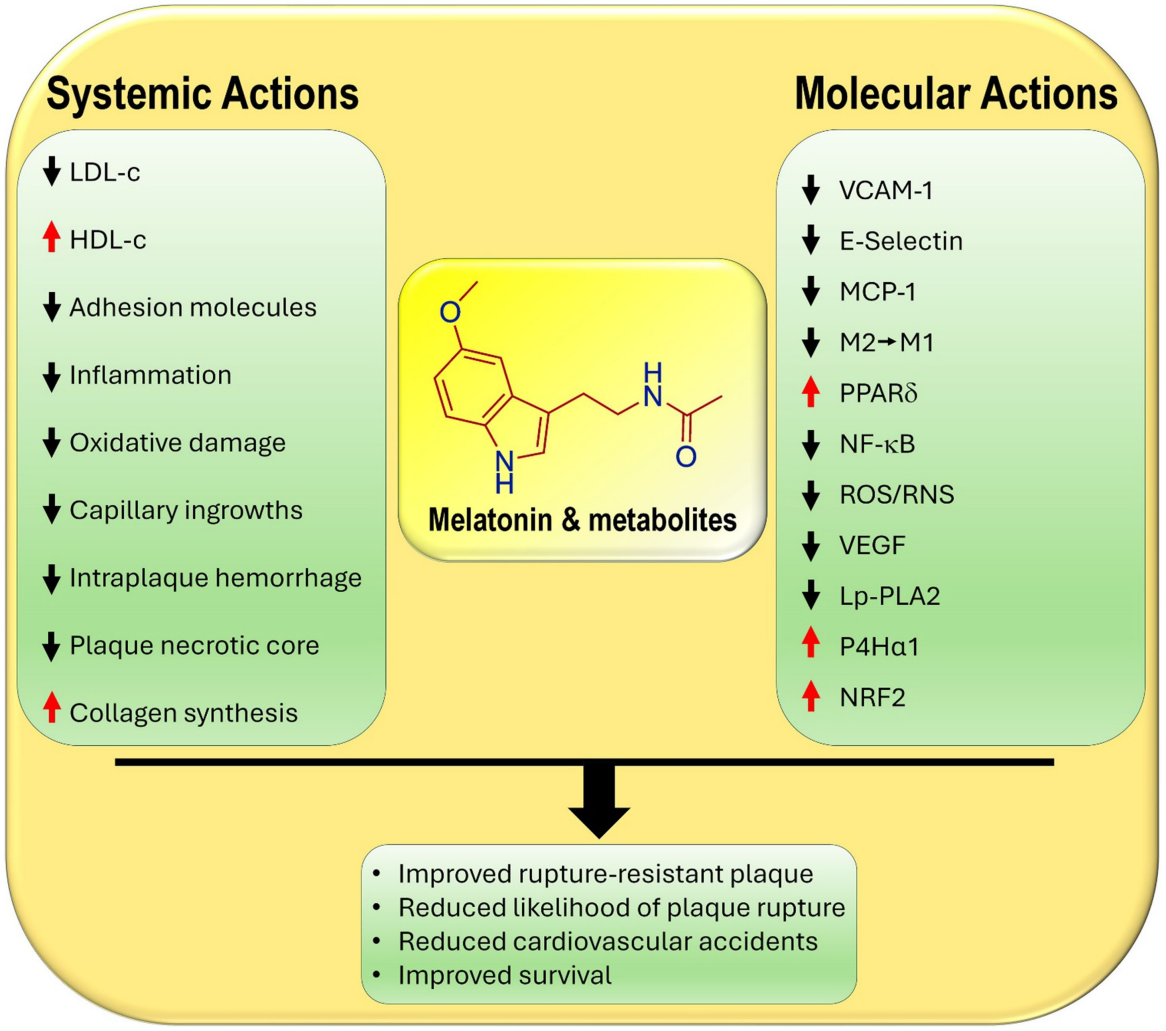
Primary and secondary known actions of melatonin and its metabolites that contribute to their ability to reduce the development and the likelihood of the rupture of atherosclerotic plaques. LDL-c, low density lipoprotein cholesterol; HDL-c, high density lipoprotein cholesterol; E-selectin, endothelial cell adhesion molecule; VCAM-1, vascular cell adhesion molecule; MPC-1, monocyte chemoattractant protein-1; M2, anti-inflammatory macrophage; M1, pro-inflammatory macrophage; PPARδ, peroxisome proliferator-activated receptor delta; NF-κB, nuclear factor kappa B; ROS/RNS, reactive oxygen species/reactive nitrogen species; VEGF, vascular endothelial growth factor; LpPLA2, lipoprotein-associated phospholipase A2; P4Hα1, prolyl-4-hydroxylase α1, collagen producing enzyme; NRF2, nuclear factor erythroid 2-related factor 2.

Another major feature of VSM cells is their high expression of the nuclear receptor and transcription factor, peroxisome proliferator-activated receptor *δ* (PPARδ), which functions in the maintenance of VSM metabolism (5). The upregulation of PPARδ interferes with aberrant lipid metabolism which is commonly associated with chronic inflammatory diseases; these include hyperlipidemia, atherosclerotic lesions and type 2 diabetes all of which are linked to plaque formation. Lien and co-workers recently published data showing that PPARδ interfered with two processes that are critical to atherosclerotic development (6); it reduced VSM cell phenotypic switching as well as endoplasmic reticulum stress-NF-κB signaling. These changes decrease the likelihood of atherosclerotic plaque rupture (Figure 1).

Li et al. published the first report that addressed the issue of the association of melatonin and atherosclerotic plaque stability (7). Initially, this group treated vascular smooth muscle cells with melatonin and examined its effect on prolyl-4-hydroxylase α1 (P4Hα1); this enzyme is essential for promoting the maturation and secretion of collagen. The molecular mechanisms involved the elevated phosphorylation of Akt and the upregulation of specific protein 1 (Sp1); Sp1 then bound to the promoter of P4Hα1, inducing enzyme expression. Chemical inhibition of Akt or Sp1 prevented the stimulatory action of melatonin on P4Hα1. An *in vivo* study was also carried out in which atherosclerotic lesions were induced in *ApoE^−/−^* mice by placing a perivascular collar around the common carotid artery to create sheer stress, leading to the development of atherosclerotic lesions. After melatonin treatment, P4Hα1 activity and collagen production were upregulated in the atherosclerotic plaques; this stabilized the caps and made them more rigid, thereby reducing their vulnerability to rupture.

In a more complete analysis of the association of melatonin with atherosclerotic plaque vulnerability, Ding et al. also used the hypercholesterolemic *ApoE^−/−^* mouse model in which rupture-prone vascular lesions were produced as a result of endogenously induced renovascular hypertension (8). Administration of melatonin orally for 10 weeks resulted in highly significant reduction in intraplaque hemorrhage and in the frequency of plaque rupture, followed by intraluminal thrombus creation. The protective actions of melatonin in reducing plaque rupture involved inhibiting inflammation by preventing the polarization of intraplaque macrophages from the M2 (anti-inflammatory) to the M1 (proinflammatory) phenotype; this conversion involved the nuclear receptor retinoid acid receptor-related orphan receptor-*α* (RORα). With the use of human monocyte-derived macrophages, these workers found that melatonin impacted macrophage polarization with the involvement of the AMPKα/STAT/RORα pathway. As with the publication by Li et al. (7), the combination of an *in vivo* and *in vitro* study by Ding and co-workers confirmed the ability of melatonin to prevent the breakdown of rupture-prone soft plaques by reducing the inflammatory response in the plaque core (8).

Atherosclerotic plaques consistently exhibit chronic and unresolved inflammation. Several processes by which melatonin represses inflammatory responses in atherosclerotic lesions have been identified. Critical agents in atherosclerosis development are oxidized LDL (ox-LDL) and a reduction in nitric oxide (NO). Melatonin reduces oxidative damage to LDL and significantly promotes NO production. Moreover, melatonin reduces the expression of genes which induce inflammation-associated pyroptosis of endothelial cells while also improving mitochondrial respiratory chain activity (9). Also, melatonin prevents the translocation of NF-κB into the nucleus and its binding to the promoter region of immunity response and inflammation genes which aids in limiting the activation of the inflammasome (1). While these actions have not been specifically documented in atherosclerotic plaques, extrapolation from other vascular sites strongly suggest they occur in atherosclerotic lesions as well.

The arterial intima is generally devoid of blood vessels; the tissue in this area receives their nutrients by diffusion from the luminal fluid. A major aspect of advanced atherosclerotic plaque development is the invasion of blood vessels from the tunica media (the vasa vasorum) into the tunica intima and subendothelial space. These new vessels in the core of the plaque proliferate, enlarging the overall lesion size; their rupture leads to intraplaque hemorrhage (IPH), which is dangerous as it puts additional pressure on the cap. Jiang et al. used a high-fat-diet (HFD), *ApoE^−/−^* mouse model in which arterial stenosis was surgically induced, with half of the animals being treated with melatonin thereafter (given daily by gavage) (10). In this report, melatonin suppressed the ingrowth into and rupture of blood vessels in carotid artery plaques. Moreover, the authors reported that melatonin, given to *ApoE*^−/−^ mice inhibited plaque progression with intraplaque growth of blood vessels being associated with melatonin-mediated activation of PPARγ and reduced RhoA/ROCK signaling. Pharmacological blockade of PPARγ restored the activity of the RhoA/ROCK pathway and aggravated the development of plaque lesions. In addition to the *in vivo* study in mice, these findings were also confirmed using human umbilical vein endothelial cells (HUVEC). This is the first report which identified that the action of melatonin on atherosclerotic plaque vascular pathophysiology involves its regulation of the PPARγ-RhoA/Rock pathway (11). The same molecular pathways influenced by melatonin *in vivo* were confirmed in an *in vitro* study using human vascular endothelial cells (HUVEC). As with other data summarized in this report, these findings suggest the therapeutic potential of melatonin in inhibiting atherosclerotic plaque maturation and rupture. The results of other studies related to potential means by which melatonin may curtail intraplaque neovascularization and aid in plaque stability were recently reviewed by Ugusman et al. (12).

Hu and co-workers examined a number of endpoints in an attempt to more precisely identify the role played by melatonin in maintaining atherosclerotic plaque stability (13). They used a HFD rabbit model in which the animals were also treated with 10 mg/kg melatonin daily for 12 weeks. At the conclusion of the treatment period, primary measurements were made using USPIO (ultrasmall superparamagnetic ion oxide)-enhanced Magnetic Resonance Imaging (MRI) to evaluate the atherosclerotic lesions in the abdominal aorta, along with a variety of biochemical analyses. Total cholesterol, LDL-c, and total triglycerides were strongly elevated as a result of HFD; these changes were significantly reversed by melatonin administration. HDL-c, the so-called good cholesterol, was increased. MRI imaging combined with USPIO, which is engulfed by macrophages, showed spotty signal voids in the aortic walls; these pathological changes were reversed in rabbits given melatonin daily. Histochemical/histological analysis showed that the cells in the aortic intima were hyperplastic and overlayed foam cells and invading vascular endothelial cells. Also, the intima-to-media thickness ratio was elevated due to the thickening of the tunica intima. All the indices measured in this study documented that the pathological changes in the vascular intima that occur during atherosclerotic plaque formation are reversed by melatonin treatment. Moreover, the findings show that the plaques that do form are of a more rupture-resistant phenotype as a result of melatonin administration.

Chen and co-workers examined in detail the role of melatonin in influencing the switching of vascular smooth muscle cell phenotype, along with the associated molecular mechanisms and their function in determining the likelihood of atherosclerotic plaque rupture (14). The changes were investigated in arterial plaques from the aortic arch of apolipoprotein E knock out (*ApoE^−/−^*), high cholesterol fed mice and in smooth muscle cells obtained from the human aorta (HASMCs). In the *in vivo* study, the mice were fed 0.3% cholesterol (high cholesterol diet: HCD) for 12 weeks beginning at 8 weeks of age; during the last 8 weeks of the 12-week treatment period, half of the mice received an intraperitoneal injection of 20 mg/kg/day melatonin. In the melatonin treated mice, the aortic plaques were smaller and the necrotic cores less extensive than in the control animals. As indices of plaque vulnerability to rupture, the collagen content, the thickness of the fibrous cap and the number of alpha-actin positive smooth muscle cells on the surface of the plaque were increased in the melatonin treated mice; these changes are all consistent with the elevated stability of the plaques with a limited probability of rupture. At the molecular level, melatonin significantly reduced switching of the smooth muscle phenotype, depressed KLF4 expression, and enhanced the upregulation of PPARδ. Silencing of PPARδ documented that this transcription factor plays a crucial role in determining smooth muscle cell switching (14). Similar changes were observed in HASMCs when they were incubated in the presence of melatonin. The authors suggested that the anti-inflammatory and antioxidative actions of melatonin in the plaque core may also have contributed to plaque stabilization. Collectively, the data provide compelling evidence that melatonin has a significant action in reducing atherosclerotic plaque destabilization by increasing the thickness of the fibrous cap and thereby reducing the prospect of plaque rupture and the associated negative downstream effects.

Lipoprotein-associated phospholipase A2 (Lp-PLA2) is an enzyme that promotes the inflammatory response; it is normally found in the blood where it is bound to LDL. This enzyme is frequently elevated in cardiovascular disease and is especially related to the development of atherosclerotic plaques (15, 16). The relevance of melatonin to Lp-PLA2 expression in atherosclerotic plaque formation was examined in HFD-fed *ApoE^−/−^* mice (17). In this model, as observed in earlier studies, melatonin elevated the content of collagen in the atherosclerotic plaques which were also reduced in size. Melatonin also provided protection against plaque generation by inhibiting Lp-PLA2 expression, reducing oxidative stress (upregulation of the NRF-2 pathway), lowering lipid peroxidation and suppressing macrophage ferroptosis (oxytosis), a type of programmed cell death that requires iron.

There are other actions of melatonin that relate indirectly to atherosclerotic plaque development. For example, melatonin attenuates the expression of vascular cell adhesion molecules (VCAM-1 and E-selectin) on endothelial cells and monocyte chemoattractant protein 1 (MCP-1), thereby reducing the adhesion of monocytes and their diapedesis into the subendothelial space (18, 19). These effects in combination with melatonin’s significant anti-inflammatory actions are critical in ameliorating endothelial damage and curtailing harmful plaque formation (20, 21) (Figure 1).

## 3 Discussion

In general, atherosclerotic plaques can be classified as soft, rupture-prone, destabilized (also referred to as low-density, non-calcified plaques) or hard, rupture-resistant, stabilized plaques (also referred to as high-density, calcified plaques) (22–25). The former are dangerous and are more likely to break down, with the core debris being released into the lumen of the vessel. The worst-case scenario occurs when the discharged molecular rubble forms a clot that interrupts the blood flow to the downstream tissues; this is especially devastating when it occurs in a vital organ such as the heart (heart attack) or brain (stroke; brain attack). Additionally, soft plaques are commonly associated with aneurysms which, when they burst, can be life-threatening (26). Rupture-resistant plaques are hard with a more rigid crown due to the accumulation of collagen, calcium and smooth muscle cells. An enlarging hard plaque also can eventually obstruct the blood flow in an artery leading to a negative cardiovascular episode; however, this is much less frequent than the rupture of a soft plaque. Clearly, the most important risk factor for the breakdown of a plaque leading to thrombosis relates to the composition of the plaque itself rather than to vascular stenosis. Thus, maintaining atherosclerotic plaques in a rupture-resistant phenotype has clear clinical advantages.

As summarized in this report, experimental data strongly suggest that melatonin functions in various ways to aid the transition of soft to hard plaques. As the data reported herein show, this conversion directly and indirectly involves melatonin’s ability to, (i), depress blood total cholesterol, LDL-c, hyperlipidemia, prevent the oxidation of LDL-c, and enhance circulating levels of HDL-c, (ii), inhibit adhesion molecules, ICAM, VCAM, and selectin, on endothelial cells, thereby lowering monocyte attachment and their diapedesis into the subendothelial space, (iii), minimize the polarization of monocyte-derived macrophages from the anti-inflammatory M2 to the proinflammatory M1 phenotype, (iv), limit the invasion of blood vessels from the tunica media into tunica intima and diminish intraplaque hemorrhage, which eases pressure on the cap and reduces the likelihood of rupture, (v), improve collagen maturation in the plaque cap hardening the plaque, (vi), suppress the overall inflammatory response and stimulate the antioxidant activity in the plaque core, and (vii) stabilize cap structure, thereby lowering the incidence of cap rupture. These observations are in line with the well documented ameliorative actions of melatonin on all aspects of atherosclerosis development (9, 27–29).

The inverse relationship of melatonin with atherosclerotic development in the aged does not prove a functional relationship; however, the fact that parentally administered melatonin unfailingly prevents/lessens plaque rupture in animal models which exhibit atherosclerotic lesions illustrates its potential usefulness at the clinical level. During aging, melatonin production wanes at a time it would likely be beneficial in preserving cardiovascular health (30, 31). Interestingly, a smaller decline in melatonin with aging seems to correlate with improved health, and vice versa. This being the case, consideration should be given to supplementing aged individuals with melatonin under a physician’s guidance, with the intent of reducing the massive physical and financial toll this condition places on the world population. This is not a totally novel idea and was already suggested nearly a decade ago (32). Currently, there is no reliable means other than perhaps dietary adjustments, which are difficult for most individuals to sustain, that have demonstrated efficacy in deferring cardiovascular deterioration during aging.

The potential importance of melatonin use for the purpose of maintaining cardiovascular health should also be seriously considered for smokers and diabetics since both groups have chronically low melatonin levels (33–35), and an elevated risk of cardiovascular disease (36, 37). Moreover, when these groups received melatonin therapy for other reasons there were improvements in their cardiac and general physiology (38–40).

Melatonin is an endogenously produced molecule that has a very long evolutionary history, having evolved perhaps 3 billion years ago, and is present in the three major domains of life: prokaryotes, eukaryotes (both plants and animals), and archaea (41). It has been found to be almost universally protective against a vast array of cardiovascular and other pathologies; its beneficial effects in many different situations seem to stem from its unusually high efficacy as an antioxidant, its potent-anti-inflammatory activity, and other receptor- and non-receptor mediated actions. Melatonin has been very widely used experimentally in cultured cells, in animals and plants and clinically in humans and domestic species, including in clinical trials (42, 43). Melatonin is widely acknowledged to be minimally toxic over a very wide range of doses (44). Side effects of melatonin in humans that have been reported include subjective estimates of headache, dizziness, grogginess, and even these are uncommon. Compared to the toxicity of other commonly used over-the-counter drugs, e, g., aspirin (gastric bleeding, death), ibuprofen (renal and hepatic damage), etc., melatonin is exceptionally safe (45–47). No documented fatalities have been reported with the exclusive use of melatonin and in experimental animals, no lethal dose (so called LD50) has been established despite attempts to do so (44). Melatonin is inexpensive and is available in pharmacologically pure form. Several idiosyncratic reactions to melatonin have been published as case reports, for example, one case each of diarrhea (48) and gynecomastia (49). The optimal or satisfactory dose to be used to resist atherosclerosis and plaque development is obvious not established since no clinical trial has tested its specific efficacy in reducing atherosclerosis or atherosclerotic plaque rupture in humans. A potential shortcoming of any study conducted would be to use too low a dose. Such intense pathological conditions such as atherosclerosis cannot be combatted with low doses of any molecule. Cardinali has seriously considered and calculated the expected dose of melatonin in human diseases (44). As a general guideline, it seems a daily dose of 0.5–1.5 mg/kg body weight would be a reasonable starting point. When melatonin is used for this purpose, it would likely be best if the treatment is started before atherosclerosis is at its advanced stages. Thus, while melatonin would be a treatment to harden plaques and reduce the likelihood of well-developed atherosclerotic plaque rupture, it would likely even be better in restricting the development of hard plaques (50). Melatonin has no known serious side effects in either the short or long term and no comparison has been done in reference to the efficacy of statins relative to melatonin in modifying plaque structure, plaque rupture or the frequency of heart attack. Finally, the statins may be more useful if combined with melatonin given that free radical mediated-oxidative stress is a contributing factor to the muscle and hepatic toxicities of these drugs, and melatonin is a well know ubiquitously acting antioxidant (50). There are no known drug contraindications for the use of melatonin. Recall, melatonin is an endogenously produced molecule in all species and every marketable drug has been tested in situations where at least physiological concentrations of melatonin exist (51).

Another reason for using melatonin to treat these pathologies relates to its chronobiological properties; circadian disruption is frequently a major obstacle for maintaining optimal health (52–54). Chronodisruption is a condition that melatonin helps to correct. While no clinical trials have been performed to specially examine the use of melatonin as an antidote for plaque rupture or heart attack, one purpose of this mini-review is to strongly encourage such studies. As a place to begin examination of the association between melatonin and atherosclerotic plaque pathologies, epidemiological studies such as that recently reported for melatonin and age-related macular degeneration (55) should be conducted.

While the data published to date provide compelling evidence that melatonin positively modifies high cholesterol atherosclerotic plaque structure, the studies have exclusively used animals (mice and rabbits). Considering the very large number of elderly patients with cardiac pathologies who already use melatonin on a regular basis (usually for sleep promotion), they would be an important cohort to examine relative to similar-aged and diseased individuals who do not use melatonin. In a sense, a trial of the efficacy of melatonin on plaque stability is already underway but is not being monitored for cardiovascular disease. Such clinically relevant comparisons should be examined with careful attention to the daily dose of melatonin, the route of administration, and duration of use relative to the severity and frequency of angina, incidence of heart attack, post-attack circulating troponin levels etc. Such studies would be most germane in the elderly where atherosclerosis is most common, nocturnal endogenous melatonin levels are known to be greatly attenuated but could be measured to provide correlative data.
